# On the Proofs of the Proximate Causes of Diseases, and More Especially of Fevers

**Published:** 1809-07

**Authors:** 


### *On the Proofs of the proximate Causes of Diseases, and more especially of Fevers; by* Dr. ALDER.

(In Continuation.)

IN my last, I promised some Reflections upon the Cases now stated. Nothing is more apt to mislead than a narrow view of facts. Some gentlemen, from seeing or reading several cases where congestions in the head were most conspicuous in those who had died from fevers, have concluded that congestion in the head, or even inflammation there, is the proximate cause of fever. Others, finding in some cases that livid blotches all over the chief internal parts, were the most striking phænomena, have concluded that repelled carbuncles caused such fevers. Others again, observing nothing very remarkable, inferred that the complaints were seated in the nerves. Others, finding much acrid bile, attributed the fevers to it; and so on. But, in order to say in what fevers do consist, we must examine impartially all the dissections which have been made after the different kinds of fevers, and this deliberately and minutely; and, at the same time, consider the symptoms of all the different kinds of fevers, the causes, and the success of the different modes of treatment. To do all this, has occupied much of my attention for more than twelve years.

From the preceding Sketch of the appearances found after all the different kinds of fever, any gentleman, however, who has not much studied this subject, may be somewhat perplexed to fix upon any one proximate cause, if he has not fully attended to what I have written. But I flatter myself, if he have adopted my idea that an imperfect transmission through the lungs is the proximate cause of fever, and then have diligently inquired into *the reasons why in some cases this does not, prima facie, appear to be so*, may find a clear clue to conduct him through all the apparent intricacies and contradictions, without very much embarrassment. In most cases, he will find every mark to lead him to conclude the lungs had been greatly congested through the whole of the disease, and that the blood, in consequence, could seldom pass freely through them from the veins into the arteries. Whenever he finds the venous system overcharged, he may be sure that the blood has not had a free passage through the lungs; and in almost all cases he will find the venous system in some parts, parts, indeed in many parts, so overcharged: but sometimes one part of it much more than another. The venous system in the liver, spleen, and head, is apt to be the most overcharged, and the soonest; but that in the stomach and intestines, and all the other viscera, often, is scarcely perceptibly behind, though it seldom, in these, is overcharged to any great extent, except the fever be very malignant or lingering: but in malignant fevers it usually is soon and greatly overloaded in all the viscera. And when fevers are lingering, chronic affections often form in the viscera, or the venous congestion reaches some remote vessels, and forms disease in them. Nothing more occurs to me at this moment by way of general reflection upon these cases; but if we descend to particulars, we shall find sufficient matter for other observations, though part of it has been anticipated.

When the patients had been copiously bled or purged, (their fever in the latter case supervening upon dysentery or a very profuse diarrhœa) the venous congestions were comparatively small; when polypi had formed during the illness in the veins going into any viscus, that viscus was not congested, but shrunk. This was sometimes the case with the lungs, as well as with the liver, and most probably was so sometimes with the head, though I do not just now particularly recollect a case of this last. The black vomit, most unquestionably, was in several cases nothing but black blood exuding from the overcharged vessels of the intestines; though in other cases, undoubtedly, it might be mixed with bile. I believe the yellowness of the skin is usually caused by absorption from the biliary pores, though I find I have been guilty of omiting some dissections which chiefly relate to this, and remember them but imperfectly.

All the common and all the uncommon symptoms of fever are well accounted for. The breathing, and parts about the chest, from the blood being arrested in the lungs, as it evidently is, may be affected in every possible variety of ways; and the appearances found on dissection, in different cases, certainly prove that they have been so. The same observations may be made upon the head, the liver, the stomach, and intestines, all the vicera, and all the parts of the body; for no part of the body can be out of the reach of disease from an impeded pulmonary transmission; and dissections shew that no part is. Every symptom of almost every disease can arise from it; and this is as much as to say, that almost every disease, whether acute or chronic, can arise from it; which is what I wish to maintain: as it is what symptoms and causes, as well as dissections and pathological reasonings, bear me out in.

Gentlemen have of late years prided themselves upon making nice distinctions among diseases, and finding out very minute differences; and this many of them would have us believe, is all that can be done to improve medicine in a *scientific way!* This, considered *in a certain point of view,* has its use. Others take diseases as they find them, and try by experiments to discover new remedies for them; and they think this is the *best* way to give to medicine any *essential improvement.* This way too is laudable. But when these gentlemen accuse others, merely because they have taken a different way from what they themselves have adopted, they assume a great deal too much; as, besides this question, which they expose themselves to, *Who is to submit to your self-erected tribunal?* it is very evident that they are interested parties, and wish to acquire a great reputation for the mode which they have adopted, at the expence of others who have pursued a different method. And if such gentlemen will but peruse the works of Hippocrates, Sydenham, Prosper Alpinus, Huxham, Cleghorn, &c. very impartially and attentively; that is, the works of every esteemed practical writer, and also the works of the systematics, they will find that the great *desideratum* in all of them is, not what those gentlemen are respectively pursuing, (though this is by no means *neglected* by them) but it is something of a very superior kind. They all, it is true, discriminate different diseases, and try to find efficacious remedies; but they all, at the same time, fill their work with facts, which can be of no use, except proper inferences be made from them. They relate the state of the seasons; the palpable causes operating both for and against the constitution; the state of peoples' body and mind; the effects of agents operating on them; the common and extraordinary symptoms, and the appearances on dissection; and they dilate upon these with accuracy and minuteness, which prove they think them matters of the utmost importance; and yet they can be but of little use, except they, by fair, extensive, and certain deductions, lead to a knowledge of the inward nature, the proximate cause of diseases, and from that to the true indications of treatment.

It is true too that these great men (so much were they struck with the importance of it) could scarcely ever restrain their their inclinations to find out the proximate causes of diseases; and if they went wrong, their endeavours, and even their errors still yield confirmation of the same great truth; for is it to be supposed that men of the first information, judgment, character and practice, would perplex themselves with pursuits, and hazard their reputation, except they conceived these pursuits were of the utmost degree of importance? Such a supposition cannot be earnestly maintained. I will grant, that if they delivered and maintained their conjectures as truths, or with any imposing manner which would make them be received with more attention than they were entitled to, they were to blame; and further, that those who built indications of treatment, or exhibited remedies conformably to them, were as much so: but still I beg it to be recollected, that we must carefully distinguish between the *principle* of a thing and the execution; and that ill success in any undertaking is not a positive proof that the *principle* of that undertaking is wrong. Though it is with sorrow that I acknowledge the common and general way of judging of the *principle* of an undertaking is by the *success of it.* Gentlemen of education, however, must see that this is a wrong way; and it is to such that I address myself. The conjectures, the hypotheses, and the theories, (as they are indulgently I think called) on medical subjects may be canvassed, and when wrong may be condemned; *nay, despised*;—but yet the contempt (however deserved) thrown on them, ought not to prejudice fair and proper inductions.

I mean to pass on now to consider what inferences are to be drawn from the success of different modes of treatment: observing only in the first place, and before I leave anatomy, that the theory I have above proposed explains *the use of the spleen!* I must confess I was not a little surprised when I found this circumstance. It is well known that the vessels in the spleen are easily distended, and may be distended to a great extent without disease or any inconveniency: hence it certainly is a receptacle for any small overflowing of the blood in the veins, intended to prevent its being thrown in considerable quantity upon some important part. The kidnies greatly assist it in this office, by draining off a great part of the fluid sent into them, and they may, in many cases, relieve the whole system more than the spleen can; but I think that in many cases, the spleen will relieve the venous system more, and particularly that part of it situated in the upper part of the abdomen; for it is well known that when the veins coming from the viscera here seated are very full, the blood coming from the spleen will *regurgitate*, and that the spleen in consequence will greatly increase in size, receiving much blood from these viscera. The spleen may, on such occasions, receive perhaps a pound of blood, and give it out again when the veins are in a condition to receive it. And if it had not received this blood, it must have pressed with violence to distend the veins situated in some of the most vital organs. This is the view which I take of the use of the spleen at present; and since writing this, I find Dr. Powel considers the liver as performing a similar office.

*Proofs from the Practice of Physic.*—A more difficult undertaking I believe is no where to be found in our profession, than to state clearly any certain and efficacious mode of practice for fevers. We find one author has succeeded in treating fevers in one way, and another another; and so on for a great variety of ways, many of them very different, and some of them, *in appearance* at least, quite contradictory. And we find others, and some indeed of the same, who have in other cases tried all these ways without any perceptible effect. Those ways, however, do no more than correspond with the great varieties which we have seen in the symptoms, and in the appearances on dissection in different fevers, and even in the same kinds and casts of fevers; the body in fever running through a great variety of states, many of them as different as have been the several successful modes of treatment, and some of them opposite. And of these different states some have predominated more in some kinds and instances of fever, and some in another. From all which circumstances it is perfectly clear that no mode of practice can be generally successful which does not make provision for, and indeed depend upon, these variations in the state of the body, not only in different fevers, but in the same kind of fever, and even in the *same case of it.* To say therefore, that opium, wine, or bark, or bleeding, or purging, or antimonials, or the cold bath, is good for fever, or for such a kind of fever, or *even for this case of it*, is in fact *very often* saying but little, even admitting it to be true, and may mislead: for the state of the body is so apt to change in fever, *that if one of these remedies be good this hour, it may not be so the next.* For instance, the cold bath is now much extolled as a sovereign remedy for fever, and by a gentleman in Jamaica, it is called a specific even for the yellow fever. It is no doubt an excellent remedy for most fevers of this country, and for many in Jamaica when when judiciously applied; that is, when used according to the directions given: but at the same time there have been and are innumerable examples of fever, and even of the yellow fever, in which it could not be used by a person with success, though he should comply strictly with the directions given. I allude to those cases of fever, in which congestions have been formed in the viscera from venous turgescence, which have run on to a low, malignant, and almost imperceptible, but nevertheless, a monstrous and fatal kind of inflammation almost in half an hour. These cases, it is true, have very seldom, of late years, been seen in this country, and they occur but seldom in the common cases of fever in most countries; yet they do occur; and I believe (taking all the fevers into view which have afflicted mankind) these casses form *at least* one half of them; leaving out all such as have had no danger in them, I believe they form two thirds; and if we contemplate only those which have been fatal, I am persuaded they have constituted much more than three-fourths. But in order to see this *clearly*, it is necessary to read the history of the most malignant forms of fever, particularly of epidemics. The appearances which I have noticed, however, as discovered by dissection, and the phenomena which I mentioned when treating of the symptoms, will I am persuaded tolerably well support me without alluding to any thing further.

*Antimony* was the great specific of Cullen; and I really, when I began to study medicine, thought that this was to remove the *spasm upon the excretories*, open them, and cure fever in almost every case. But I find that in most fevers it is a pernicious medicine. It is however to be depended on in some; viz. in those which have some topical inflammation joined with them; and in this respect, it is in some measure an exception to the remark I before made, as it will give great relief *in such cases*, without any very *great* nicety as to the time of its administration.

*Wine* too has been cried up as a sovereign remedy in fever. But I am persuaded that, *for general practice*, the exhibition of this ought to be limited by as many "provisos and exceptions" as any remedy whatever.

I would here proceed to lay down certain indications of treatment as resulting from my theory, and see how the successful modes of practice which have been used, correspond with them: but I find I have no room to make much progress in this business in this letter; I shall therefore reserve it to be the subject of my next. But I wish to notice notice now, that various reports have got about, concerning *a conspiracy to insult the Profession and the University of Edinburgh;* but I declare I know of no such conspiracy: as to the profession I have a great regard for it; and with respect to the University, and the students at it, I have a great respect for them too; but surely gentlemen cannot generally know, what is no where taught; and teachers cannot teach it without they discover it by their own laborious exertions. It is not the practitioners, nor the teachers of medicine which I complain of; *but the science and the practice of it.* I have said, that the present race of teachers and practitioners in medicine is much more enlightened than any which has gone before: but if notwithstanding this, I were to add even that the science and the practice of physic are yet to be brought to certainty and efficacy, (if they can be brought to it) I persuade myself I should fall in with the convictions of every well-informed medical man.

I believe, however, the reflections above alluded to, were levelled chiefly at Dr. Beddoes (for his late letter) and the society of medical reformers; probably, without grounds. For myself, I am sure the body of practitioners could give me very essential aid in the immediately following part of my undertaking; and at the same time I am also inclined to fear, that without some such sort of assistance, it must come forth very far short of what I could wish.

( To be continued. )

